# Antibody-Based Sensors: Principles, Problems and Potential for Detection of Pathogens and Associated Toxins

**DOI:** 10.3390/s90604407

**Published:** 2009-06-05

**Authors:** Barry Byrne, Edwina Stack, Niamh Gilmartin, Richard O'Kennedy

**Affiliations:** 1 Centre for Bioanalytical Sciences (CBAS), Dublin City University, Dublin 9, Ireland; E-Mail: Barry.Byrne@dcu.ie (B.B.); 2 National Centre for Sensor Research (NCSR), Dublin City University, Dublin 9, Ireland; E-Mails: Edwina.Stack3@mail.dcu.ie (E.S.); Niamh.Gilmartin@dcu.ie (N.G.); 3 Biomedical Diagnostics Institute (BDI), Dublin City University, Dublin 9, Ireland

**Keywords:** pathogen, antibody, biosensor, electrochemical, surface-plasmon resonance, assay development

## Abstract

Antibody-based sensors permit the rapid and sensitive analysis of a range of pathogens and associated toxins. A critical assessment of the implementation of such formats is provided, with reference to their principles, problems and potential for ‘on-site’ analysis. Particular emphasis is placed on the detection of foodborne bacterial pathogens, such as *Escherichia coli* and *Listeria monocytogenes*, and additional examples relating to the monitoring of fungal pathogens, viruses, mycotoxins, marine toxins and parasites are also provided.

## 1. Introduction

Pathogenic bacterial, fungal and viral cells are ubiquitous in nature and pose a considerable risk to human and animal health, in addition to severely compromising the quality of agricultural produce (Table 1). Therefore, the monitoring of these microorganisms is of paramount importance for the prevention of nosocomial infections, the maintenance of general public health and for ensuring compliance with legislative and quality standards. The rapid detection and identification of a pathogen is essential, in particular where food samples with short shelf-lives are being analysed, or where the urgent administration of a suitable antimicrobial agent is required to treat a potential fatal infection. Virulent pathogens may often be present in low numbers in samples, demonstrating that high sensitivity and specificity are also absolute necessities. Hence, developing suitable detection methods which permit accurate, rapid and sensitive analysis is essential for monitoring the distribution of pathogens and, most importantly, ensuring customer/patient safety.

**Figure 1.**
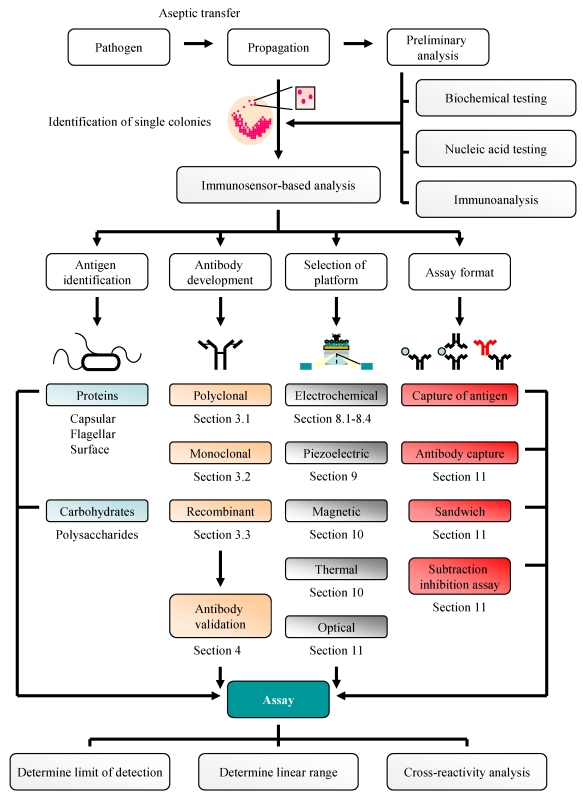
Strategy for pathogen detection.

This review provides a comprehensive summary of the principles, problems and potential of using immunosensor-based analytical platforms for pathogen detection. It describes the development of electrochemical, potentiometric, piezoelectric and optical platforms for the monitoring of foodborne bacterial pathogens by incorporating monoclonal, polyclonal or recombinant antibodies in a variety of different assay formats. The overall strategy adapted is shown in Figure 1. The analysis of fungal cells, associated toxic secondary metabolites, viral and water-borne pathogens (toxins and parasites) is also outlined. Finally, the advantages of using sensor-based methodologies as an alternative to more traditional methods of pathogen detection, namely bacteriological testing and nucleic acid-based analysis, and alternative sensor-based formats (e.g. biomimetic and plant sensors), will be discussed.

## 2. Bacteriological and Nucleic Acid-Based Analysis of Pathogenic Bacteria: A Traditional Approach

The culturing of pathogenic and non-pathogenic prokaryotic strains involves the aseptic transfer of an innoculum from a source (soil, food etc.) to suitable growth medium which results in amplification of microbial cell numbers, subsequently permitting quantitative determination [1]. This propagation may be performed in the presence of selective markers, such as antibiotics, to suppress the growth of other strains that may also reside in the innoculum. Subsequent transfer to selective or differential media generates colonies that can be distinguished based on their distinctive colony morphologies by ocular inspection (Table 2) and their identification confirmed by rigorous biochemical (glucose utilisation etc.) or nucleic acid-based assays [2].

Colony count estimation provides an inexpensive and user-friendly protocol for quantitative and qualitative bacterial pathogen detection, and one which is routinely employed in the development of hazard analysis and critical control point (HACCP) systems within the food industry and for the establishment of risk assessments [2,7]. However, a major disadvantage of this approach is the lengthy times required to obtain visible colonies that can be identified. This may take up to 7 days for *L. monocytogenes* cells, cultured using the NF EN ISO 11290-1 protocol [3,8], and over 2 weeks for another important food-related pathogen, *Campylobacter fetus* [9]. Further complications with using this methodology arise from the ability of some bacterial strains to be viable but non-culturable. This phenomenon, and its importance from the perspective of the food industry, has recently been discussed with reference to *L. monocytogenes* [10] and *E. coli* O157:H7 [11].

An alternative method for pathogen detection, and one which is often used in conjunction with active culturing to provide sufficient biomass, involves the amplification and subsequent analysis of pathogen-specific nucleic acid by polymerase-chain reaction (PCR) and sequencing (Table 3). The versatility of these methodologies is emphasised by the ability of real-time PCR to provide rapid data analysis of multiplex PCR to facilitate the simultaneous analysis of multiple pathogens and of reverse-transcriptase PCR to differentiate between viable and non-viable cells. Furthermore, the presence of bacterial RNAs (mRNA and tmRNA) in food samples can be determined through the use of nucleic-acid sequence-based amplification (NASBA) [12,13]. However, the implementation of these methodologies for pathogen detection can be complicated by external factors. For example, strains may originate from complex sample matrices, e.g. food sources that often contain high levels of fats, carbohydrates and other entities which necessitate a sample clean-up stage prior to analysis. Furthermore, as discussed by De Boer and Beumer [7], the amplification of nucleic acid from a pathogenic strain is indicative only of its presence in the sample of interest and cannot be used to monitor toxin production qualitatively or quantitatively. Non-specific DNA amplification may also be observed; the presence of ‘naked’ DNA in analytical samples may act as a template for the amplification of these superfluous products [14] which complicates fingerprint-based analysis. Therefore, alternative methods of pathogen analysis (e.g. antibody-based) can be more useful.

## 3. Antibodies: Production and Purification

**Figure 2.**
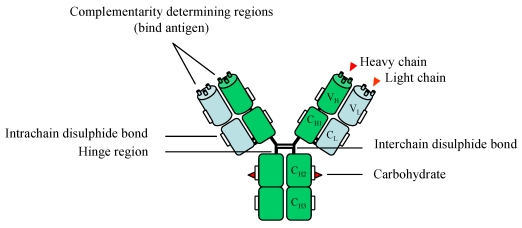
A schematic representation of an IgG antibody comprising of two heavy (green) and light (blue) chains. Carbohydrate elements are attached via the asparagine 297 amino acid residue. A more in-depth discussion of antibody glycosylation is provided in reference [30]. Key: V_H_ – variable heavy, V_L_ – variable light, C_H_ – constant heavy, C_L_ – constant light.

**Figure 3.**
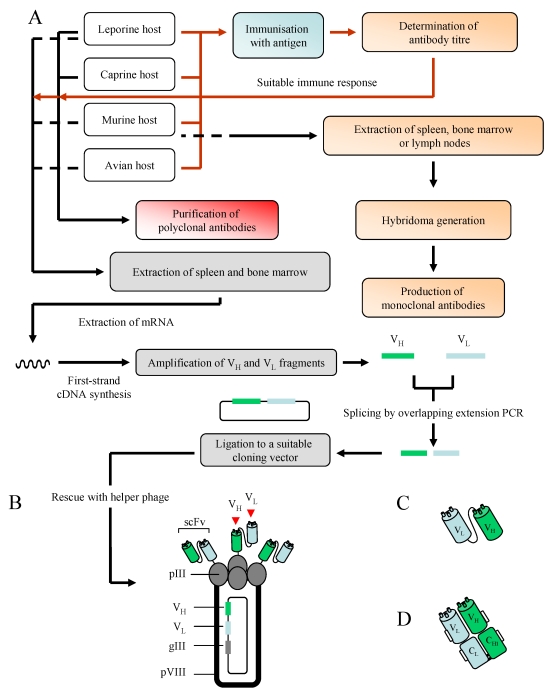
An overview of monoclonal, polyclonal and recombinant antibody production [A]. Immunisation-related stages are represented by a red line, with those involving antibody production shown in black. A more in-depth discussion of the generation of recombinant antibodies, inclusive of Fab fragments, can be found in reference [38]. Additional hosts may also be used for antibody production, including camels (camelid), sheep (ovine) and pigs (porcine). A filamentous phage displaying scFv antibody fragments [B] and two recombinant antibody fragments, the scFv [C] and Fab [D], are also illustrated. Key: pIII/pVIII – protein 3/8, V_H_ – variable heavy, V_L_ – variable light.

A schematic representation of a full-length antibody is shown in Figure 2. Polyclonal, monoclonal and recombinant antibodies have frequently been selected for a wide variety of applications, including immunodiagnostics and biomarker detection. Their production involves the exploitation of the immune system of murine, leporine, ovine or avian hosts (Figure 3A). For the production of bacterial pathogen-specific antibodies, these hosts may be immunised with cells which may [28] or may not [6] be heat-treated (exceptions include naïve antibody phage display libraries which are constructed independently of immunisations; see below). These antigens are typically administered in the presence of a suitable adjuvant, and the immune response generated by the host after a series of immunisations can be determined by screening serial serum dilutions for recognition of the antigen in an enzyme-linked immunosorbent assay (ELISA)-based format.

### 3.1. Polyclonal Antibodies

Polyclonal antibodies (pAb) are typically raised in rabbits, goats or sheep [29], and their popularity is illustrated by the fact that they are frequently selected in immunosensor-based assays for pathogen detection. It should be stressed that the inherent nature of pAbs means that a selection of different epitopes may often be recognised on a single cell. In cases where this is undesirable, such as in the case where high specificity is a requirement, monoclonal or recombinant antibodies may be more applicable.

### 3.2. Monoclonal Antibodies

Monoclonal antibodies are generated through the use of hybridoma technology [31,32] and murine hosts are commonly selected for immunisation. The bone marrow, primary lymph nodes and, most commonly, the spleen are selected as a source of antibody-producing B cells which are harvested and fused to immortal myeloma cells. The resulting hybrid cells (referred to as hybridomas) subsequently secrete full-length antibodies that are directed towards a single epitope. Suitable candidates, identified by ELISA-based analysis, are then ‘cloned out’ to ensure that a single cell, producing antibody specific for an individual epitope, is present and the antibody generated can be used for assay development.

### 3.3. Recombinant Antibodies

Recombinant antibodies, generated through the use of phage display technology and the biopanning of antibody repertoires (libraries) against a target of interest [33,34], have been selected for the detection of a range of structurally diverse antigens, including proteins [35], haptens [36] and carbohydrate moieties [37]. Three types of library may be used as sources of antibody pools; namely synthetic, naïve and immune. Synthetic libraries are produced by PCR-based randomisation of complementarity-determining regions (CDR) from heavy and/or light chains, and naïve antibody libraries are typically assembled from B-cells extracted from unimmunised human donors. Immune libraries are constructed from RNA isolated from spleenocytes or the bone marrow of a host (avian, murine, leporine etc.) immunised with an antigen that generates the required immune response. The RNA acts as a source of complementary DNA (cDNA) which, in turn, serves as a template for the amplification of variable heavy (V_H_) and variable light (V_L_) gene sequences which can be fused through an overlapping-extension splicing PCR reaction and subsequently cloned into a suitable phage or phagemid vector (Figure 3A) [note that in the case of fragment antigen binding (Fab) construction (see below), this process involves the fusion of variable and constant regions]. The introduction of this construct into suppressor strains of *E. coli* (such as XL1 Blue) by electroporation, in conjunction with the packaging of phage particles via the addition of helper phage (a process referred to as rescuing), allows the encoded antibody structure to be ‘presented’ on the exterior of a bacteriophage particle, as illustrated in Figure 3B. Two types of antibody fragments may be presented, namely the single-chain variable fragment (scFv) and the Fab, and these are illustrated in Figure 3C and 3D. The production of these fragments is dependent on the vector selected for harbouring the library [38].

Biopanning is used for the selection of binders from an antibody library which may contain between 10^7^ and 10^10^ different antibody-encoding gene sequences. To achieve this, the antigen is immobilised on solid phase (e.g. on a column or immunotube) or bead-conjugated (in solution phase) and the antibody pool is subjected to recurrent rounds of selection against the antigen with increasing levels of stringency in terms of binding ability. Selected binders are retained and subjected to additional screening to increase their specificity for the target (affinity maturation), which can be monitored by ELISA-based analysis. The production of soluble antibody fragments can be facilitated by infecting phage pools into non-suppressor *E. coli* strains, such as Top-10F' or HB2151, and inducing with isopropyl-β-D-1-thiogalactopyranoside (IPTG) in the presence of low concentrations of glucose. These hosts recognise the amber (AUG) codon engineered between the scFv and gIII gene [39], producing scFv or Fab fragments independent of the phage coat proteins.

The majority of the examples given in this review involving immunosensor-based pathogen detection incorporate monoclonal or polyclonal antibodies. However, recombinant antibodies are as yet not fully exploited in this field and have several significant advantages over conventional antibodies. The specificity and sensitivity of recombinant antibodies for a particular antigen can be significantly enhanced by the targeting of CDR regions using site-directed mutagenesis or chain shuffling [40,41]. Further advantages include the capacity to incorporate tags (e.g. His or C-myc) for isolation and, subsequently, immobilisation, the ability to fuse various labels (e.g. green fluorescent protein or enzymes) directly to the antibody fragment facilitating and simplifying detection, and the availability of a range of antibody formats (e.g. scFv, Fab, re-engineered IgG, dimers etc.). Avian hosts, in particular, have been shown to be useful for the production of high-affinity recombinant antibodies [35,42,43].

## 4. Antibody Selection

When selecting monoclonal, polyclonal or recombinant antibodies for the detection of pathogens, certain characteristics are of great importance. Firstly, the antibody should be able to detect and quantify very low cell numbers (sensitivity) and this may frequently be an issue for foodborne-related bacterial pathogens (Table 2).

Secondly, it should be able to differentiate specific strains of interest from related microflora which may also reside in the sample (specificity). Hence, the selection of a highly-specific epitope on the pathogen is a key consideration, since many bacterial strains share homologues of surface-presented proteins which can lead to the detection of multiple cell-types by a single antibody. It is therefore recommended that a constitutively-expressed antigen, which is species-specific, is targeted [3]. Where possible, the expression of this target antigen should not be highly dependent on the growth matrix of the pathogen. Finally, the antibody should bind with its cognate target with sufficient strength to permit interrogation (high-affinity). The identification of an antibody candidate that satisfies these requirements can be facilitated through screening by ELISA-based analysis to reduce the number of potential antibodies to a smaller number which can subsequently be screened by sensor-based analysis to identify the candidate with the best affinity for the target epitope. This antibody can then be further selected for incorporation on an immunosensor-based platform.

## 5. Use of Antibodies for Antigen Isolation/Enrichment Prior to Analysis

Antibodies can be successfully used to isolate and collect pathogens from complex matrices where numbers are low and the sample volume is large. Immunomagnetic separation (IMS) involves the coating of a pathogen-specific antibody on a magnetic bead which can be used to facilitate binding, concentration and removal of pathogenic cells from a complex sample media (provided that the antibody has sufficient specificity). Retrieved cells can then be propagated on selective of differential media. The versatility of this technique is further illustrated by the ability to detect pathogen-antibody complexes with beads coated with a cognate secondary antibody, an indirect assay format demonstrated by Torensma and co-workers [44] for the detection of *L. monocytogenes* cells bound to a murine monoclonal antibody produced from the immunisation with whole bacterial cells. IMS has also been applied for the detection of these cells in cheese [45], *S. typhimurium* in bovine faecal matter [46] and *E. coli* O157:H7 in beef carcasses [20,47] and bovine faeces [48].

## 6. ELISA and Microarray-Based Pathogen Detection

ELISA-based analysis can be directly applied for the detection of foodborne pathogens. Brooks and co-workers [9] developed an ELISA-based assay for the detection of *C. fetus* in bovine preputial washing and vaginal mucous samples. A sandwich assay format was developed by Kerr *et al.* [49] for the detection of a selection of different *E. coli* O157:H7 strains from human and animal subjects by using a monoclonal antibody specific for a fimbrial antigen. The limit of detection was similar to that found by Brooks *et al.* (1 × 10^5^ CFU/mL). ELISA-based assays have also been described for the detection of *L. monocytogenes* [50,51] and *Salmonella enterica* spp. [52]. A major drawback of these assay formats is that analysis times are often lengthy. Typical ELISA assays are comprised of a number of steps; namely blocking, washing, incubation of primary and secondary antibodies and substrate development. These can take several hours to complete and, understandably, this may be problematic in instances where rapid detection is a requisite.

Protein microarrays are excellent candidates for high-throughput analysis of biomolecular interactions in miniaturised assays formats [53]. The implementation of antibodies on such platforms for pathogen detection offers a flexible approach for the screening of high number of bacterial isolates from numerous sample matrices. Array formats typically consist of a panel of pathogen-specific antibodies spotted onto individual positions on a microarray slide by dedicated robotic handling (printing), with subsequent pathogen detection commonly employing sandwich ELISA assay formats. Gehring and colleagues [54] printed a biotinylated antibody (caprine-derived) for *E. coli* O157:H7 on a streptavidin-coated microarray slide. Captured cells were further probed with a fluorescein-labelled secondary antibody and microarray spots were visualised through the use of fluorescent microscopy. This sandwich assay format had a linear range of detection of 3 × 10^6^ - 9 × 10^7^ cells/mL. Cai *et al.* [55] developed an antibody microarray capable of the parallel analysis of somatic (O) and flagellar (H) antigens on 117 *Salmonella* strains from twenty commonly encountered serovars, including *typhimurium, heidelberg* and *enteritidis*. Thirty five polyclonal antibodies from rabbit antiserum were spotted in pairs on commercially obtained microarray slides and used for the capture of Eosin Y-fluorescently labelled cells. Over 73% of the strains selected for analysis were positively identified, with an additional 30 strains partially serotyped. The ability of this assay format to selectively differentiate between related and unrelated *Salmonella* strains was demonstrated by the analysis of an additional 73 strains selected from a panel of almost forty non-target serovars. Anjum *et al.* [56] also targeted O-antigen groups of 17 *E. coli* strains with rabbit polyclonal antisera. The ability of these microarray formats to screen numerous bacterial pathogens in parallel was further illustrated by the printing of individual antibodies in a 96-well polystyrene plate by an ‘in-house’ robotic printing system. This cost-effective array format, devised by Gehring and colleagues, was selected for the simultaneous detection of *E. coli* (1 × 10^6^ cells/mL) and *Salmonella typhimurium* (1 × 10^7^ cells/mL) in buffer and ground beef extract [57] and further illustrates the versatility of such antibody-based formats. Finally, antibody-based microarray platforms have been selected for the sensitive and parallel detection of structurally diverse pathogens. These include bacterial strains that pose a potential bio-terrorism risk (e.g. *Burkholderia mallei, F. tularensis* and *Y. pestis*) and viral particles (e.g. West Nile virus) [58], foodborne pathogens (*Campylobacter jejuni*) and mycotoxins [59] and, finally, spore-forming bacterial cells (*B. globigii*) and toxins [60]. The latter assays [59,60] implement the Naval Research Laboratory (NRL) array biosensor. This elegant platform can simultaneously detect pathogenic bacterial cells and toxins, and can perform sandwich (as is the case with many of these examples) and competition immunoassays in parallel with an assay time of approximately 15 minutes [59]. Toxin and virus-related pathogen detection is discussed in sections 13 and 14 with reference to immunosensor-based analysis.

## 7. Biosensors

**Figure 4.**
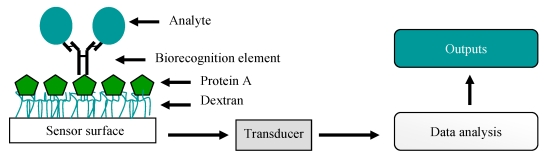
A simple representation of a biosensor. Here, a full-length antibody is captured on protein A immobilised on a carboxymethylated dextran-coated sensor surface and is used for the capture of an analyte. This interaction produces a specific physicochemical change, such as a change in mass, temperature or electrical potential. This is then converted (via a transducer) to a signal which the user can interpret.

Biosensors are analytical devices which combine a biological recognition ligand with physical or chemical signaling devices (transducers). The recorded biomolecular interactions are transformed into digital signals which are interpreted by a computer-aided readout, thereby providing the user with a representation of the interaction that occurs between the bound (ligand) and free (analyte) entities (Figure 4). Many different sensor formats have been utilised for pathogen analysis using antibodies; namely electrochemical, mass-based, magnetic and optical. The sensitivities of these assays are dependent on the properties of the transducer and the quality of the antibody. An overview of each sensor type and an explanation of how antibodies can be incorporated for pathogen detection follows.

## 8. Electrochemical Immunosensors

The principle behind these assay formats is the coupling of a specific antibody with an electrode transducer which functions to convert a binding event into an electrical signal. In general, electrochemical biosensors can be based on four transducer types; namely amperometric, impedimetric, potentiometric and conductimetric.

### 8.1. Amperometric Platforms

Many amperometric biosensors utilise an enzyme-based system that generates an electroactive product which can be oxidised or reduced at a working electrode (carbon, gold etc.). The resultant current can then be detected. This format has several advantages, including the capacity to fabricate disposable and customised screen-printed electrochemical electrodes (screen-printed carbon electrodes) by depositing inks (carbon, silver etc.) in a pre-determined arrangement and thickness [61]. These systems are economical, robust and sensitive and can be used in conjunction with mediators such as ferrocenedicarboxylic acid (FEDC) or iodine to improve selectivity [3]. Furthermore, there is major potential to miniaturise these systems. This leads to smaller sample volume requirements.

Gehring *et al.* [62] developed an amperometric assay for the detection of *S. typhimurium* cells which were captured with magnetic bead-conjugated antibodies and detected with an alkaline-phosphatase (AP)-labelled goat anti-*Salmonella* antibody. After deposition of the beads on graphite ink electrodes, the AP-catalysed production of electroactive *para*-aminophenol (PaP) from its substrate, *para*-aminophenyl phosphate (pAPP), was monitored electrochemically and the generated signal was directly proportional to the number of captured bacterial cells. This assay had a sensitivity of 8 × 10^3^ cells/mL. Ivnitski and co-workers [63] also applied this methodology for the detection of *Campylobacter*. Here, anti-*Campylobacter* antibodies were embedded in a bilayer lipid membrane and, upon binding with free cells, a conformational change was introduced which allowed the transport of ions through the membrane. The resultant current was detected amperometrically via a stainless steel electrode. This rapid (10 minutes) assay allowed the researchers to verify that 10^10^ ions could pass through the channel per second. This value was correlated with a theoretical value of one bacterial cell per sample. Lin and co-workers [64] recently immobilised a monoclonal antibody on screen-printed carbon electrodes (SPCE) for the capture of pure cultures of *E. coli* O157:H7, and implemented a horseradish peroxidase (HRP)-labelled polyclonal antibody for detection in an indirect sandwich assay format. It was noted that the attachment of gold nanoparticles and the use of FEDC, as a mediator, resulted in a noticeable amplification of the response current generated. This enabled the detection of approximately 5 × 10^3^ CFU/mL in 1 hour. The assay had excellent selectivity and specificity, with minimal cross-reactivity observed when groups of other food pathogens were tested in parallel (*L. monocytogenes, Salmonella choleraesuis* and *Vibrio paraheamolyticus*), thus illustrating the importance of having a selective biorecognition element. Crowley and colleagues [65] also selected a SPCE-based platform for *L. monocytogenes* detection. A direct sandwich assay format, consisting of a leporine polyclonal capture antibody and an AP-labelled detection antibody, could detect 9 × 10^2^ cells/mL. Comparable sensitivity was observed when polyclonal goat (1 × 10^3^ cells/mL) andrabbit (9 × 10^2^ cells/mL) antibodies were used for capturing cells in an indirect sandwich assay format. The direct immobilisation of *L. monocytogenes* cells on the SPCE provided a low response.

### 8.2. Impedimetric Platforms

Impedimetric biosensors are often based on the fact that the metabolic redox reactions of microorganisms are detectable and quantifiable when performed in the presence of a suitable mediator [66]. Hence, viable microbial biomass can be determined by monitoring microbial ‘metabolism’ which, in turn, increases conductance and capacitance and results in a decrease in impedance. Similarly to amperometric biosensors, several elegant antibody-based impedimetric assays have been used for pathogen detection. Radke and Alocilja [67] developed a high-density microelectrode array for the sensitive detection of *E. coli* O157:H7 (1 × 10^4^ – 1 × 10^7^ CFU/mL) using a goat anti-IgG polyclonal antibody for capture. Tully and colleagues recently implemented a biotinylated leporine polyclonal antibody for the detection of internalin B (InB), a *L. monocytogenes* cell-surface protein. When captured on avidin-coated planar carbon electrodes modified with polyaniline, a conductive polymer, the limit of detection for InB was found to be 4.1 pg/mL [68]. The versatility of using this approach for the detection of this bacterium was also illustrated by Wang *et al.* [69] who adopted a different protocol by immobilising a monoclonal antibody on a titanium-dioxide nanowire to detect 1 × 10^2^ CFU/mL. Finally, Su and Li [70] demonstrated how a quartz crystal microbalance (QCM) system using impedance could detect *S. typhimurium* in chicken meat. The implementation of magnetic beads resulted in a significant improvement in assay sensitivity, with a limit of detection of 1 × 10^2^ cells/mL. Minimal cross-reactivity was observed with *E. coli*.

### 8.3. Potentiometric Platforms

In potentiometric biosensors the conversion of a biorecognition process into a change in potential signal is detected by a reference electrode. Potentiometric biosensor formats typically consist of a perm-selective outer layer and a bioactive element, such as urease, may be introduced to enhance the performance of the assay [3]. A methodology that combines potentiometric and optical detection, namely the light-addressable potentiometric sensor (LAPS), was shown to be applicable for pathogen detection. Gehring *et al.* [71] implemented this technology in conjunction with an immune-ligand assay (ILA) for the detection of *E. coli* O157:H7. The assay format devised involved the enumeration of cells by biotinylated polyclonal capture and fluorescein-labelled detection antibodies which were raised in caprine hosts through the administration of heat-killed cells. This ‘sandwich’ complex (in the presence of an additional urease-labelled anti-fluorescein antibody) was subsequently captured on a streptavidin-bovine serum albumin (BSA)-coated nitrocellulose membrane. Urease enzymatic activity was monitored by the hydrolysis of urea to carbon dioxide and ammonia. The authors were able to detect approximately 7.1 × 10^2^ cells/mL and 2.5 × 10^4^ cells/mL of heat-killed and live cells of *E. coli*, respectively, in buffered solutions. Dill and co-workers [72] utilised a silicon chip-based LAPS assay to detect low levels (119 CFU) of *S. typhimurium*. Here, biotinylated and fluorescein-labelled anti-*Salmonella* antibodies were selected as biorecognition elements. This assay format was subsequently applied for the monitoring of chicken carcass washings spiked with *Salmonella* cells, and demonstrated a high recovery rate for cells (90%).

### 8.4. Conductimetric Platforms

The final electrochemical immunosensor format that will be discussed, with reference to the detection of *E. coli* and *Salmonella* spp., is based on conductimetric detection [73]. Here, a biological signal is converted to an electrical signal via a conductive polymer, such as polyacetylene, polypyrrole or polyaniline. Muhammad-Tahir and Alocilja [74] developed a conductimetric biosensor incorporating a polyclonal antibody-based sandwich assay format in which the detection antibody was labelled with polyaniline. This sensor could detect approximately 79 CFU/mL and 83 CFU/mL of *E. coli* O157:H7 and *Salmonella* spp., respectively. This approach was also used for the detection of *E. coli* cells in a selection of different sample matrices, including lettuce and strawberries [75]. The sensitivity recorded was 81 CFU/mL. Furthermore, the assay was rapid (6 minutes) and could be generated in a disposable format.

Hnaiein and co-workers [76] developed a novel conductimetric immunosensor for *E. coli*. A biotinylated polyclonal antibody was captured on streptavidin-coated magnetite nanoparticles. These were subsequently bound on a conductimetric electrode through the use of glutaraldehyde coupling. Conductimetric measurements were facilitated through the application of an alternating-current (ac) voltage. The incorporation of nanoparticles facilitated an increase in conductivity, enabling 0.5 CFU/mL to be detected. A small amount of background was observed when *S. epidermis* cells were assayed in parallel. This was attributed to the use of a polyclonal capture antibody and reinforces the view that in some assay formats, monoclonal or recombinant antibodies may be more suitable.

## 9. Mass-Based Immunosensors

Piezoelectric biosensors operate on the principle that a change in mass, resulting from the biomolecular interaction between two entities, such as an antibody and its respective antigenic determinant, can be determined [77]. For example, in a quartz crystal, mass changes result in alterations in resonance frequency. Piezoelectric immunosensors are affordable and disposable options for pathogen detection, and the implementation of QCM for the direct detection of analytes, such as bacterial cells, alleviates the need for labelled secondary antibodies [78]. Babacan and co-workers [79] demonstrated that the use of protein A for the capture of a polyclonal antibody to *S. typhimurium* enhanced reproducibility and surface stability when compared to polyethyleneimine-glutaraldehyde (PEI-GA) coupling. The resultant assay format permitted the detection of 1.6 × 10^9^ CFU/mL.

Fung and Wong [80] described how the use of ethyl-N′-(3′dimethylaminopropyl)-carbodiimide hydrochloride (EDC) and N-hydroxysuccinimide (NHS) coupling, a methodology routinely selected for the immobilisation of ligands on optical sensor platforms, allowed the capture of a monoclonal antibody for *S. paratyphi* A. The use of this surface immobilisation chemistry was shown to provide good stability and sensitivity, with a limit of detection of 1.7 × 10^2^ cells/mL. With respect to both of these formats and previously mentioned assays involving protein A immobilisation [70], the selection of a proper strategy for correctly orientating antibodies is conducive to enhanced sensitivity and selectivity. Kim and co-workers [81] used a QCM platform based on impedance measurement for the detection of *S. typhimurium* (the limit of detection was approximately 1 × 10^3^ CFU/mL). Su and Li [78] developed a piezoelectric sensor for detecting between 1 × 10^3^ to 1 × 10^8^ CFU/mL of *E. coli* O157:H7 through the implementation of antibodies on a QCM via a 16-mercaptohexanedecanoic acid (MDHA) monolayer. Pohanka *et al.* [82] used a polyclonal antibody linked to the piezoelectric crystal surface using glutaraldehyde to detect *E. coli*. The resulting assay was rapid, permitting analysis in ten minutes (inclusive of a regeneration step for re-analysis), and greater than ten assays could be performed without the need for re-calibration. This ‘label-free’ assay had a limit of detection of 1 × 10^6^ CFU/mL.

## 10. Thermometric and Magnetic Immunosensors

In therometric biosensors thermistors are frequently selected as temperature transducers [83]. Magnetic biosensors, in contrast, implement magnetic beads coated with a suitable ligand that can be detected within a magnetic field. From the perspective of bacterial pathogen detection, the latter platforms have been explored to a greater degree than their thermometric counterparts. Magnetic systems offer distinct advantages. For example, when a sample selected for analysis does not contain any contaminating materials with magnetic properties, background signals (non-specific) are minimised. Ruan and colleagues [84] immobilised anti-*E. coli* antibodies on a magnetoelastic cantilever through the construction of a self-assembled monolayer (SAM). The principle of this assay was the conversion of a substrate, 5-bromo-4-chloro-3-indolyl phosphate (BCIP), to an oxidised and insoluble blue precipitate via an AP-catalysed reaction (secondary antibody). This product accumulated on the sensor surface, and the resulting changes in resonance frequency were recorded, facilitating the detection of 1 × 10^2^ cells/mL of *E. coli* O157:H7. Mujika *et al.* [85] recently developed a magnetoresistive sensor for the detection of *E. coli*. It consisted of a sandwich assay whereby the bacterial cells were captured with a polyclonal antibody and detected using leporine polyclonal antibodies coated on superparamagnetic beads. The application of an external magnetic field was used for monitoring. This assay had a sensitivity of 1 × 10^5^ CFU/mL of *E. coli* O157:H7. Furthermore, minimal cross-reactivity was seen when *S. choleraesuis* was tested on this format. With reference to immobilisation strategies, when comparative analysis was performed between three different materials, silicon nitride was found to be more suitable than silicon dioxide (SiO_2_) and SU-8 for antibody capture. Finally, this sensor format was hand-held, and these miniaturised formats demonstrate one approach for ‘on-site’ pathogen detection.

In conclusion, electrochemical, piezoelectric and magnetic immunosensors can all be applied to foodborne pathogen detection. Optical platforms also offer a powerful and ‘label-free’ methodology that permits ‘real-time’ pathogen detection, and these are discussed in section 11.

## 11. Optical Immunosensors

**Figure 5.**
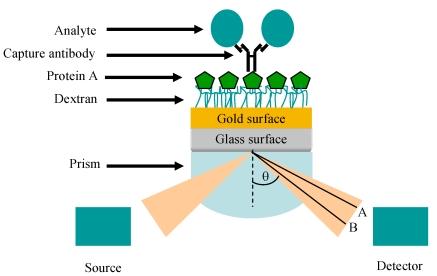
Representation of the SPR phenomenon, showing the Kretschmann prism arrangement originally proposed in references [86] and [87]. For illustrative purposes, a protein-A (green hexagon)-captured IgG antibody is shown on a carboxymethylated dextran (CM5) sensor surface. The mass change introduced by the binding of an analyte of interest (blue circle) is shown as a change in refractive index (A to B) which can be determined through the use of dedicated software.

Surface-plasmon resonance (SPR) is a phenomenon that results from the illumination of a metallic surface, such as gold, by visible or near-infrared radiation from a monochromatic light source via a hemispherical prism which exits to a detector (photodiode array) at an angle related to the refractive index (RI). The resultant oscillation of free electrons generates surface plasmons (electromagnetic waves) which resonate and absorb light. The specific wavelength/angle at which this occurs is a function of the RI in the proximity of the gold surface and relates to the mass on the chip surface. A change in mass, effected by the immobilisation of a ligand and, subsequently, further interactions which take place when analytes are passed over the modified sensor surface, causes a shift in the resonance to a longer wavelength and, hence, introduces a refractive index change (Figure 5).

A large selection of commercially available optical biosensors can be directly applied for pathogen detection. Wei *et al.* [88] used the SPREETA™ SPR system (Texas Instruments) for the detection of *Campylobacter jejuni*. Here, biotinylated leporine polyclonal antibodies were immobilised directly on the sensor surface and the assay had a sensitivity of 1 × 10^3^ CFU/mL. Barlen and co-workers [89] selected the Plasmonic SPR device (Plasmonic Biosensoren) for the detection of *Salmonella typhimurium* (2.5 × 10^5^ CFU/mL) and *S. enteritidis* (2.5 × 10^8^ CFU/mL). Mazumdar and colleagues also selected the same biosensor system for the detection of *S. typhimurium* (1.25 × 10^5^ cells/mL) in milk by implementing leporine polyclonal capture and detection antibodies [90]. A range of other optical sensor platforms, including the ProteOn XPR36 (Bio-Rad) and SensíQ (Nomadics) and Biacore™ (discussed below) also have the potential to be applied for pathogen monitoring. Oh *et al.* [91] devised a SPR-based protein chip assay format with immobilised monoclonal antibodies against *S. typhimurium, E. coli* O157:H7, *Yersinia enterocolitica* and *Legionella pneumophila*. 1 × 10^5^ CFU/mL of each pathogen could be specifically detected with their respective antibody.

Koubová *et al.* [92] were able to detect 1 × 10^6^ CFU/mL of *L. monocytogenes* and *S. enteritidis* on an ‘in-house’ SPR system, while Taylor *et al.* [93] devised an eight-channel SPR sensor for permitting the detection of *E. coli* O157:H7 (1.4 × 10^4^ CFU/mL), *L. monocytogenes* (3.5 × 10^3^ CFU/mL), *C. jejuni* (1.1 × 10^5^ CFU/mL) and *S. choleraesuis* (4.4 × 10^4^ CFU/mL) in buffer (PBS). Rijal and colleagues [94] applied a novel fibre-optic biosensor for the detection of *E. coli* O157:H7 by immobilising a monoclonal antibody on a silanised (3-aminopropyl-triethoxysilane, APTES) silica fibre-tapered surface using EDC/sulpho-NHS coupling. Changes in light transmission (470 nm) were introduced by pathogen binding, and the assay had a sensitivity of 70 cells/mL. Alternative fibre optic-based platforms that use fluorescent detection include the Analyte 2,000 [95] and the RAPTOR biosensor. The latter is a portable device that utilises a sandwich ELISA format for detecting pathogens. Typically, a biotinylated capture antibody is immobilised on an avidin-coated fibre-optic waveguide. Four such channels are housed within a plastic disposable ‘coupon’, thereby permitting parallel analysis to be performed with four different analytes. Detection antibodies are labelled with a fluorophore, typically cyanine 5 (Cy5) [96] or Alexa fluor 647 (AF647) [97]. Fluorescently-tagged molecules that are located within 100 – 1,000 nm of the waveguide surface are excited by a diode laser (635 nm), and a percentage of the emitted fluorescence is detected by an optical probe and quantitated by a photodiode detector that collects emitted light at wavelengths of over 650 nm [96]. The RAPTOR biosensor has been used for detecting foodborne pathogens, including *S. typhimurium* in spent water samples of spiked alfalfa seeds [98], *L. monocytogenes* in frankfurter meat [99] and *Enterococcus faecalis* from recreational water samples [100]. Pathogens can also be recovered and propagated by incubating waveguides containing bound bacterial cells in selective media post-analysis [98].

These examples demonstrate the use of commercial and ‘custom-built’ SPR systems. A more detailed discussion of Biacore-based analytical approaches will now be provided, together with the problems encountered with these assay formats and methods for overcoming them.

**Figure 6.**
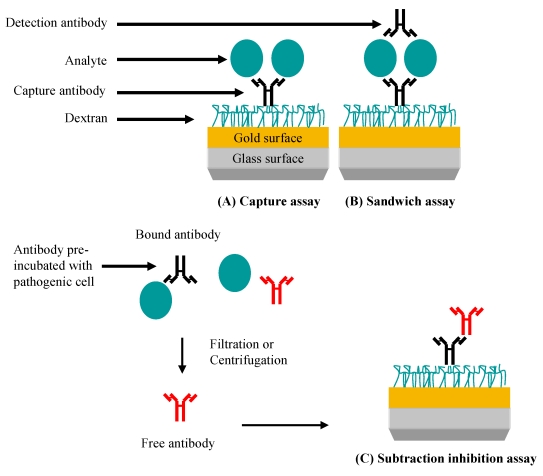
SPR-based assays for pathogen detection. (A) Specific antibody is immobilised and is used to capture the pathogen leading to a signal. (B) Pathogen or pathogen-related antigen is captured. Specificity is conferred by the binding of a second antibody. (C) Specific antibody reacts with the pathogen or pathogen-related antigen. Non-bound (free) antibody is isolated and detected when bound to an immobilised antibody (normally an anti-species antibody) on the chip. In this case, the signal generated is inversely proportional to the pathogen concentration.

The versatility of Biacore-based analytical platforms is demonstrated by the ability of the researcher to perform capture, sandwich or subtractive-inhibition assays, as shown in Figure 6. Hearty and colleagues [6] produced a murine monoclonal antibody which was shown to be specific for the surface-located *L. monocytogenes* internalin A (InA) protein in native and recombinantly-expressed forms. When this antibody was immobilised on a CM5 surface through EDC/NHS coupling, a limit of detection of 1 × 10^7^ CFU/mL was observed when *L. monocytogenes* cells were tested. Cross-reactivity studies clearly demonstrated the specificity of this monoclonal antibody, with minimal binding to *E. coli, B. cereus* and *Listeria innocua* (the latter selected due to the non-expression of the InA protein) observed. This further illustrates the importance of this antibody as a species-specific reagent.

Sandwich assay formats are routinely selected for increasing sensitivity in ELISA-based analytical platforms. This format was adapted for SPR-based analysis of *E. coli* O157:H7 and *Salmonella* by Fratamico and colleagues [101]. They demonstrated that the sensitivity of a capture assay for *E. coli* O157:H7 cells (5 × 10^9^ CFU/mL) could be enhanced significantly by the subsequent addition of a caprine polyclonal antibody, which enabled the detection of between 5 and 7 × 10^7^ CFU/mL.

Another interesting observation deduced from this experimental work related to the immobilisation strategy. The initial experimental format implemented a capture assay format (5 × 10^9^ CFU/mL). No apparent increase in sensitivity was observed when protein A was used to immobilise the mAb. The ability of a sandwich assay format to enhance sensitivity was also described by Bokken *et al.* [102] for the detection of *Salmonella* groups B, D and E. The original capture format permitted the detection of 1 × 10^7^ CFU/mL. This sandwich format used a monoclonal capture and polyclonal detection antibody, the former immobilised through standard EDC/NHS coupling. This assay format reduced the limit of detection to 1.7 × 10^5^ CFU/mL. While these assays clearly illustrate the potential that sandwich formats have for increasing assay sensitivity, it should also be mentioned that this is not always successful, as shown by the inability of two anti-*L. monocytogenes* polyclonal antibodies to enhance the signal in an assay format where cells were originally captured by a mouse monoclonal antibody [6]. There are also additional concerns with using this sandwich format on Biacore-based platforms due to the large size of the bacterial cells which exceeds the penetration depth of an evanescent wave (see section 15).

The subtraction inhibition assay (SIA) is an extremely useful method for pathogen detection in SPR-based immunoassays, and can be selected in instances where the user does not want to expose the system to pathogenic cells or to matrices which may have high viscosities. The principle of this assay format involves pre-incubating an antibody with a target pathogen and separating free from bound antibody. The quantity of free antibody is inversely proportional to the concentration of pathogen. Haines and Patel [103] implemented this assay for the quantification of *Salmonella*. A polyclonal antibody (specific for cell-wall epitopes) was incubated with freshly-prepared cells and subsequently passed through a syringe filter (0.22 μm), enabling unbound antibody to be separated from antibody-pathogen complexes. Free antibody was then captured on an anti-Fab-coated CM5 Biacore chip. This novel assay format permitted five different strains to be detected at similar sensitivities (1 × 10^4^ CFU/mL), and allowed comparative analysis with an additional ten unrelated strains at high concentrations (1 × 10^8^ CFU/mL) to be performed. No response of any statistical relevance was observed, illustrating the versatility of the SIA assay format to be used for selective analysis. Leonard *et al.* [28] developed a SIA assay for *L. monocytogenes* but adopted a different approach. A polyclonal antibody, produced through the immunisation of a rabbit with heat-treated cells, was purified by protein G affinity chromatography and added to differing concentrations of heat-killed cells in phosphate buffered saline solution (PBS) and incubated at 37°C for 20 minutes. A centrifugation step was used as an alternative to filtration for the separation of free polyclonal antibody, and subsequent analysis was performed on a goat anti-rabbit polyclonal antibody immobilised on a CM5 surface. The efficacy of this assay format was illustrated by the low limit of detection (1 × 10^5^ cells/mL), and by the short assay time required to obtain data (30 minutes; excluding preparation of the sensor surface).

## 12. Immunosensor-Based Assays for the Detection of Other Bacterial Pathogens

A selection of immunosensor-based analytical platforms has also been developed for the detection of other bacterial pathogens, including *Yersinia pestis, Vibrio cholerae, Mycobacterium tuberculosis* and *Brucella abortus* (Table 4). Furthermore, an increase in public concern has resulted from the elevated numbers of nosocomial infections which have been caused by bacterial strains such as *Clostridium difficile* and methicillin-resistant *S. aureus* (MRSA). The latter bacterial strain produces 17 enterotoxins [2] and several immunosensor platforms have enabled the detection of *Staphylococcal* enterotoxin B (SEB). Harteveld and co-workers [104] developed a piezoelectric immunosensor for detecting 0.1 μg/mL of SEB through the development of a competition assay.

The capture of a rabbit polyclonal antibody on the sensor surface did not permit the detection of free SEB. The researchers, therefore, immobilised the toxin and subsequently passed over varying concentrations of antibody pre-incubated with the toxin. The response generated was inversely proportional to the concentration of free antigen in solution. A rapid (less than 10 minutes) fibre-optic SPR-based assay was also developed by Slavík *et al.* [105], capable of detecting 10 ng/mL of SEB. Immobilisation of antibodies (polyclonal) was facilitated by glutaraldehyde coupling. Moreno-Bondi and colleagues [106] reported that it was possible to detect femtogram (fg) quantities of human serum antibodies against SEB using an array biosensor (other antigens detected in this study included diphtheria toxin, hepatitis B surface antigen (HBsAg) and tetanus toxin), while a SPR-based assay, reported by Subramanian *et al.* [107] permitted the detection of whole *S. aureus* cells in direct and sandwich assay formats (1 × 10^5^ CFU/mL). The sensor format used in this assay was the Reichert SR7000, with alkane monothiol and dithiol dendritic tether-based SAMs used for the capture of polyclonal antibodies via EDC/NHS coupling.

## 13. Immunosensors for Fungal Pathogens and Mycotoxins

The detection of fungal strains is of great importance, due to their association with crop spoilage and their involvement as human pathogens, and immunosensor-based technologies have been developed for their determination.

A key consideration with analysing fungal cells relates to the fact that they are significantly larger than their bacterial counterparts, with fungal spores often over 40 micrometers in diameter [119]. This is understandably problematic in terms of system blockage. Hence, SIA assays, described earlier for bacterial strains [28,103], may be used to circumvent this problem.

Skottrup *et al.* [120] pre-incubated a murine monoclonal antibody with sporangia of *P. infestans* at 37°C for 1 hour. The separation of bound and unbound antibody was permitted by a five minute centrifugation (1500g). Free antibody was then passed over a surface containing bound goat-anti-mouse polyclonal antibody, allowing quantitation of free antibody. The limit of detection was 2.2 × 10^6^ sporangia/mL, and no cross-reactivity was seen when other fungal strains, such as *Melampsora euphorbia* and *Botrytis cinerea* were assayed in parallel. A similar assay was also used to detect spores of *Puccinia striiformis*, using a polyclonal rabbit anti-mouse IgM antibody to capture a mouse monoclonal IgM antibody produced from immunisation with whole urediniospores. Completion of the resultant assay took approximately 45 minutes with a detection level of 3.1 × 10^5^ urediniospores/mL [121]. Additional immunosensor formats have also been used for the detection of human fungal pathogens. Muramatsu *et al.* [122] applied a piezoelectric sensor for detecting *Candida albicans* through the immobilisation of an anti-*Candida* antibody on a palladium-plated electrode. The recording of a loss in resonance frequency enabled the detection of 1 × 10^6^ CFU/cm^-3^. Medyantseva and colleagues [123] targeted an antigenic determinant on *Trichophyton rubrum* with a polyclonal antiserum. Their amperometric immunoassay had a sensitivity of 1 × 10^-15^ mg/mL of antigen and was rapid (20 minutes).

**Figure 7.**
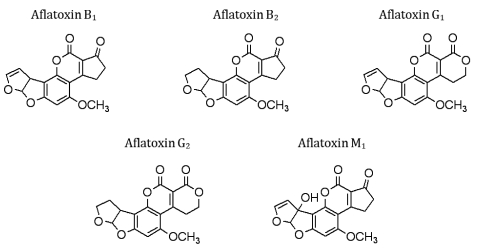
Structures of commonly encountered aflatoxins.

The monitoring of the presence of aflatoxins, naturally occurring mycotoxins produced by several strains of *Aspergillus spp.*, in fruit, vegetable and food produce, is also of great significance. Aflatoxins can cause contamination of nuts (almonds, walnuts), cereals (rice, wheat, maize) and oilseeds (soybean and peanuts). While approximately 16 structurally diverse aflatoxins have been reported, aflatoxins B_1_, B_2_, G_1_ and G_2_ and M_1_ (Figure 7) represent the greatest danger to human health [124]. Daly *et al.* [125] used a rabbit polyclonal antibody to detect AFB_1_, which was conjugated to BSA and immobilised onto a CM5 Biacore chip. A competition assay between free and bound AFB_1_ permitted a linear range of detection of trace levels (3 – 98 ng/mL). Daly and co-workers [126] subsequently generated murine scFvs against AFB_1_ by using a phage display format and incorporated these antibodies into a Biacore-based inhibition assay. Dunne *et al.* [127] also developed a SPR-based inhibition assay that incorporated monomeric and dimeric scFv antibody fragments for permitting the detection of between 390 and 12,000 ppb and 190 and 24,000 ppb of AFB_1_ with monomeric and dimeric scFvs, respectively. Adányi *et al.* [128] developed an optical wavelength lightmode spectroscopy (OWLS)-based assay for the detection of AFB_1_ and ochratoxin A. Integrated optical wavelength sensors were used in conjunction with murine monoclonal antibodies, with the sensitive detection range for a competitive assay being between 0.5 and 10 ng/mL. An indirect screening protocol was subsequently applied for the detection of these toxins in wheat and barley samples.

## 14. Immunosensor Assays for the Detection of Viral Pathogens, Marine Toxins and Parasites

The versatility of immunosensor-based analytical platforms for pathogen detection is further illustrated by the ability to develop assays for the sensitive detection of viral pathogens (Table 5). These include Hepatitis-C virus, the severe acute respiratory syndrome (SARS) virus and bovine diarrhoeal virus, whose particles present an additional selection of structurally diverse epitopes which can be targeted by antibodies. Single-celled phytoplankta play important roles in the aquatic environment by providing nourishment for a selection of heterotrophic marine animals. These include filter-feeding bivalve molluscs, such as mussels, clams and scallops. Among the reported 5,000 species of marine phytoplankton, 300 have been postulated to occur in high-numbers, causing harmful algal blooms (HABs) or ‘red-tide’ events. Approximately 40 of these species produce secondary metabolites, collectively referred to as phycotoxins [129], that are structurally diverse and non-proteinaceous compounds which have low molecular weights (in contrast to whole cell pathogens). They pose a considerable risk to human health by causing respiratory, neurological or gastrointestinal problems at low concentrations. The primary route of infection is through the ingestion of contaminated shellfish meat or drinking water. Furthermore, HABs also have a devastating effect on the shellfish industry and algal blooms can also result in reduced tourist activity and concomitant economic losses. Several countries have established regulations and specific concentration limits for phycotoxinlevelsin seafood [130].

**Figure 8.**
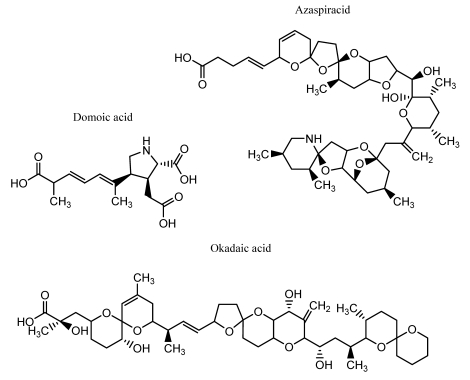
Structures of commonly encountered phycotoxins.

Phycotoxin groups are classified according to the associated symptoms of infection, and a selection of structures is shown in Figure 8. Paralytic shellfish poisoning (PSP)-associated toxins are water soluble, thermostable tetrahydropurine molecules which are subdivided into four structural categories, namely carbamate, *N*-sulphocarbamoyl, decarbamoyl and dideoxycarbamoyl. The most commonly encountered PSP-causing toxins are gonyatoxin (GTX) and saxitoxin (STX). The latter is especially toxic, and over 20 structural analogues with differing degrees of toxicity have been reported in nature [144]. A causative agent of amnesic shellfish poisoning (ASP) is domoic acid, a potent kainoid neuro-excitatory toxin which is synthesised by the marine diatom *Pseudo-nitzchia pungens* [145] and functions by binding to specialised receptors and inducing depolarisation of neuronal cells.

Diarrheic shellfish poisoning (DSP) originates from the consumption of shellfish material contaminated with the polycyclic ether toxins okadaic acid (OA), dinophysis-toxin 1 (DTX1) and pectenotoxins (PTX). Yessotoxin (YTX) is also classified under this grouping as it was isolated in 1987 from scallops associated with a DSP-related poisoning event. However, it was noted that the pharmacological properties of YTX differed from those of DSP toxins [146]. Okadaic acid, DTX and PTX are all produced by dinoflagellates belonging to the *Dinophysis* and *Prorocentrum* species, whereas YTXs are synthesised by *Protoceratium reticulatum* [147]. Finally, cyanobacterial poisoning is caused by the hepatoxins microcystin (MC) and nodularin during red-tide events. Several bacterial species have been identified as being causative agents, including members of the geni *Microcystis, Anaebaena* and *Planktothrix* and consumption of contaminated water supplies is the primary route of infection [148].

A small number of immunosensor-based formats for the monitoring of phycotoxins have been developed (Table 6), and these have mainly focussed on BTX, DA, MC, OA and STX. Several factors have contributed to this low number. A key factor is the scarcity of sufficiently pure toxin for antibody generation and the poor supply of reference material for assay development [149]. This has been problematic in the development of immunosensor-based assays for detecting other important marine toxins.

Azaspiracid (AZP) shellfish poisoning was first reported in 1995 in the Netherlands and was attributed to the consumption of mussels (*Mytilus edulis*) which were originally cultivated in Killary harbour, Ireland [163]. A total of 27 congeners of AZP have been characterised [164], and the producing strain has been postulated to be the dinoflagellate *Protoperidinium* spp. [165]. No antibodies have been developed against this target in its natural state, although an ELISA using an ovine polyclonal antibody against a synthetic AZA hapten was reported [166]. The availability of more reference material should permit additional assays to be developed for this and other marine toxins. Another important aspect for antibody-based marine algal toxin detection relates to the structural similarity that exists between toxin congeners. Furthermore, if a mixture of toxins is analysed in an immunosensor format, underestimation or overestimation of toxicity may occur as a result of an antibody being able to recognise multiple isomers of the same toxin molecule. This suggests that suitable antibody candidates have to be rigorously screened to ensure that cross-reactivity does not occur. Finally, biosensors for marine toxins should permit the detection of a toxin in a complex matrix, such as shellfish meat. The formats described in Table 6 were developed in an attempt to replace the current regulated methods of marine toxin detection, including the mouse bioassay and high-performance liquid chromatography-mass spectrometry (HPLC-MS). It remains to be seen whether they will be incorporated into legislation or routine monitoring programmes in the near future.

Immunosensor-based assay formats have allowed the detection of a selection of water-borne parasites. A piezoelectric assay was described by Campbell and Mutharasan [167] for the detection of between 100 and 10,000 oocysts/mL of *Cryptosporidium parvum*, while Kang and co-workers [168] developed a Biacore-based immunosensor assay which allowed the detection of between 1 × 10^2^ – 1 × 10^6^ oocysts/mL. The flexibility of using this methodology has also been illustrated by the ability to also detect other parasitic pathogens, including *Schistosoma japonicum* [169-172] and *Borrelia burgdorferi* [173], which act as causative agents of schistosomaisis and lyme borreliosis, respectively. These assays use amperometric [169-171], piezoelectric [172] and optical [173] -based platforms.

## 15. Antibody-Based Biosensors: Potential Issues

This review has outlined the principles and practices of antibody-based sensors for facilitating the detection of bacterial, fungal and viral species and toxins. A wide range of different applications have been highlighted involving the use of polyclonal and monoclonal antibodies (and, to a lesser extent, recombinant antibodies). However, it should also be emphasised that several problems may need to be addressed when developing related sensor-based assays, and these are now discussed.

Several of the aforementioned assays have also focused on a particular antigen. While this is also the most effective method for ensuring specificity, this may also be detrimental in instances where the exposure of a bacterial strain to stress, such as osmotic shock, alterations in pH or temperature fluctuations, or different growth media (e.g. different food matrices) may compromise the expression of a selective antigen. Hahm and Bhunia [174] exposed cells of *L. monocytogenes, Salmonella* spp. and *E. coli* O157:H7 to a variety of stress conditions and noted that antibody-based responses were reduced. Hearty and colleagues [6] heat-treated *L. monocytogenes* cells and assayed these alongside untreated cells on a monoclonal antibody-immobilised Biacore surface. A significant decrease in signal was observed when cells were treated at 60°C for 10 minutes, an observation putatively attributed to an alteration in the topography of the bacterial cell wall introduced by this treatment. These observations postulate that the sensitivity of an antibody may be compromised by an external factor, reiterating the importance of bacteriological propagation considerations. This point is particularly important in cases where antibodies are unable to differentiate between viable and non-viable cells, with active culturing able to circumvent this problem.

Several of the assays described in this review have been performed on SPR-based analytical platforms, and have involved the detection of bacterial and fungal cells whose sizes are typically between 1 – 5 micrometers and in excess of 40 micrometers, respectively [119]. Capture formats are typically used, involving the immobilisation of an antibody and the subsequent capture of a cell and, if deemed necessary, the addition of secondary antibody to enhance sensitivity [89,101,102]. In Biacore-based analytical systems, the depth at which a SPR-produced evanescent wave can penetrate when TIR occurs is 0.3 μm [28,101]. Hence, the direct immobilisation of large bacteria and fungal cells, whose diameters exceed this area, might compromise detection. Conversely, in the cases where bacterial cells are captured on immobilised antibodies, the whole bacterial cell will not be contained within this area, implying that only a portion of the cell will be able to contribute towards a RI change. This observation may explain why shorter dextran chain lengths, such as those selected by Bokken *et al.* [102] (F1 or CM3 Biacore sensor chips) may be more suitable as, in theory, the bacterial cell is spatially arranged closer to the surface and, hence, is more exposed to the evanescent wave field.

It is also worth noting that Biacore detection systems typically monitor SPR angles on the sensor surface over an area of 0.25 mm^2^ [101]. This implies that a reduced SPR signal may arise from large cells sterically hindering each other if present in large amounts. This problem can be addressed by monitoring the sensor surface by atomic force microscopy (AFM), as discussed by Hearty *et al.* [6] who were able to undock a CM5 chip, containing *L. monocytogenes* cells bound to a monoclonal antibody, incubate overnight in a glutaraldehyde-cacodylate buffer and fix in the presence of osmium tetroxide. Dehydrated chips, treated with ethanol, could then be analysed to monitor surface topography. Finally, it is worth mentioning that ELISA and Biacore-based assays differ from each other in that the former typically involves a ‘static’ incubation of antibody and pathogen, while Biacore, and indeed several other assay formats, have additional considerations, including fluid forces. It is therefore of great importance that the antibody selected has sufficient affinity to allow cells to be captured and, most importantly, retained to permit further analysis [28]. This limitation effect can be overcome thorough the use of low flow-rates, such as 1 μL/minute [6].

## 16. Alternative Sensor-Based Platforms for Pathogen Detection

Biomimetic sensors (e.g. ‘electronic noses’ and ‘electronic tongues’) and plant sensors can be selected as alternative methodologies to immunosensors for detecting pathogens. Electronic noses are comprised of sensor arrays that are capable of detecting a selection of compounds (e.g. ketones, aldehydes, aromatic and aliphatic compounds) produced during the growth stages of bacterial strains on a certain substrate. Needham and colleagues [175] were able to detect one bacterial (*B. subtilis*) and two fungal strains (*Penicillium verrucosum, Pichia anomala*) on bread before visible signs of spoilage were observed. Lipoxygenase-based enzymatic spoilage could also be differentiated from microbial spoilage, and this methodology was coupled with gas chromatography-mass spectrometry (GC-MS) for characterisation of the ‘volatiles’ (e.g. 1-butanol, 2-butanone) produced during growth of these strains. Alocilja *et al.* [176] were able to differentiate strains of *E. coli* O157:H7 from unrelated *E. coli* strains by monitoring the gaseous products produced when cells were propagated in a nutrient broth liquid culture. The electronic nose-based sensor chamber incorporated four metal-oxide gas sensors for the detection of volatile products of *E. coli* growth, such as amines, ketones and alcohols. This investigation allowed the researchers to demonstrate that *E. coli* O157:H7 had a different gas signature pattern from the unrelated strains tested in parallel. Furthermore, Balasubramanian and co-workers were able to detect *S. typhimurium* in spiked vacuum-packed beef striploins (2.6 CFU/g beef) [177]. In contrast, electronic tongues focus on the analysis of liquid samples, and are applicable for the analysis of food quality [178]. This biomimetic sensor format was selected by Lan and colleagues for the detection of *S. typhimurium* (1 × 10^6^ CFU/mL) in chicken carcass samples [179].

Non-antibody biomimetic receptor molecules, including engineered proteins, peptides, aptamers (single stranded DNA or RNA), ribozymes or synzymes (synthetic enzymes), also have potential in the detection of pathogens and other food contaminants [180]. A piezoelectric biosensor using oligopeptides designed to mimic the binding site of the aryl hydrocarbon receptor (dioxin receptor) protein was used to sensitively detect dioxins (1 – 20 ppb) [181]. Similarly, surface-immobilised antimicrobial peptides (e.g. polymyxins B and E) were used to detect *S. typhimurium* (5 × 10^4^) and *E. coli* O157:H7 (1 × 10^5^ CFU/mL) in direct and sandwich assay formats [182]. Pan *et al.* [183] reported the successful detection of *S. enterica* serovar *Typhi* by using a single-stranded RNA aptamer (S-PS_8.4_) that bound to pili (type IVB) expressed on the bacterial cell that were instrumental in promoting pathogenesis.

The ability of plants sensors (phytosensors) to detect environmental conditions and plant pathogens is still in its infancy in terms of sensor technology. A phytosensor capable of detecting plant pathogens at the molecular level was described by Mazarei and co-workers [184]. Transgenic tobacco plants, containing an inducible plant defense mechanism linked to the β-glucuronidase reporter gene, inoculated with *Alfalfa mosaic virus* showed increased β-glucuronidase expression.

These examples demonstrate that the combination of synthetic receptors mimicking nature with desired transducers can be selected as an alternative to immunosensor-based analysis for pathogen detection, although further development will be needed before these alternative formats are selected above immunosensor platforms for pathogen analysis.

## 17. Conclusions

The importance of antibodies as biorecognition elements for pathogen detection was discussed. Antibody-based sensors can provide robust, sensitive and rapid analysis. In most cases the key element is the quality of the antibody used and recombinant antibodies have many advantages, including the ability to be genetically modified to improve selectivity, sensitivity and immobilisation. In practice, the development of these assays is simplified through the development of a suitable antibody and, subsequently, an assay format. While there are several problems associated with these methods, the potential for monitoring bacterial, fungal, viral and parasitic pathogens is immense.

Innovative recent developments, such as the hand-held device described recently by Mujika *et al.* [85], signal the way forward for pathogen detection. Future trends will continue to implement immunosensor-based technologies into microdevices, ultimately permitting on-site analysis to be performed in a rapid, reliable and sensitive manner.

## Figures and Tables

**Table 1.** A selection of pathogenic bacterial, fungal and viral strains and their virulence traits.

**Pathogen**	**Pathogenic trait**
**Bacterial Pathogens**	
*Bacillus anthracis*	Human pathogen; causative agent of anthrax; toxin producer
*Bacillus subtilis*	Putative human pathogen: causative agent of food poisoning
*Brucella abortus*	Human and animal pathogen; causative agent of brucellosis
*Campylobacter* spp. and *C. jejuni*	Human pathogen; causative agent of campylobacteriosis
*Clostridium botulinum*	Human pathogen; producer of neurotoxins and causative agent of botulism
*Escherichia coli* O157:H7	Human pathogen; causative agent of foodborne illness and producer of toxins, such as verocytoxin or ‘shiga-like’ toxin
*Francisella tularensis*	Animal pathogen; putative biohazard
*Legionella pneumophila*	Human pathogen; causative agent of Legionnaires disease (legionellosis)
*Listeria monocytogenes*	Human pathogen; causative agent of listeriosis
*Mycobacterium tuberculosis*	Human pathogen; causative agent of tuberculosis
*Neisseria meningitidis*	Human pathogen; causative agent of bacterial meningitis
*Salmonella typhimurium*	Human pathogen; causative agent of salmonellosis
*Staphylococcus aureus*	Human pathogen; causative agent of hospital-acquired infection, toxin producer
*Yersinia enterocolitica*	Human pathogen; causative agent of yersiniosis
*Yersinia pestis*	Human pathogen; potential causative agent of the black plague
**Fungal pathogens**	
*Candida albicans*	Human pathogen; causative agent of vaginal thrush
*Puccinia striiformis*	Plant pathogen; causative agent of stripe rust
*Phytophthora infestans*	Plant pathogen; causative agent of potato blight
*Trichophyton rubrum*	Human pathogen; causative agent of athlete's foot and ringworm
**Viral pathogens**	
African swine fever virus	Animal pathogen; causative agent of African swine fever
Bovine diarrhoea virus	Animal pathogen; causative agent of mucosal erosion and bovine diarrhoea
Cowpea mosaic virus	Plant pathogen; causes mosaic pattern, vein yellowing and leaf malformation
Ebola virus	Human pathogen; causative agent of severe haemorrhagic fever disease
Foot and mouth virus	Animal pathogen; causative agent of acute degenerative disease in cattle
Hepatitis C virus	Human pathogen; causative agent of blood-borne infectious disease
Human immunodeficiency virus	Human pathogen; causative agent of acquired immunodeficiency syndrome (AIDS)
Rift valley fever virus	Animal pathogen; causative agent of Rift valley fever
SARS-associated coronavirus	Human and animal pathogen; causative agent of severe acute respiratory syndrome
Tobacco mosaic virus	Plant virus; causes mottling and discolouration of leaves
West Nile virus	Human and animal virus; causative agent of West Nile fever and encephalitis

**Table 2.** Three commonly encountered bacterial foodborne pathogens with their selective media and epidemiological relevance. Figures obtained for annual estimated cases and infectious doses (*) are obtained from reference [3] and are representative of figures calculated by the United States Department of Agriculture (USDA) economic research service. Key: CFU - colony-forming units.

**Strain and morphology**	**Selective media**	**Clinical signs of infection**	Estimated annual cases *	Infectious doses (CFU) *
***E. coli* O157:H7** Gram negative rod	Cefixime rhamnose sorbitol MacConkey agar [4] SEL media [5].	Diarrhoea (bloody) Renal failure Haemolytic uraemic syndrome (rare)	173,107	1 × 10^1^ - 1 × 10^2^
***Salmonella* spp.** Gram negative rod	Bismuth sulphide agar [4] SEL media [5]	Cramps Diarrhoea Vomiting	1,342,532	1 × 10^4^ - 1 × 10^7^
***L. monocytogenes*** Gram negative rod	*Listeria* enrichment broth [4,6] Fraser broth [4] SEL media [5]	Vomiting Abdominal cramps Fever	2,493	400 - 1 × 10^3^

**Table 3.** A selection of nucleic acid-based protocols for pathogen detection.

**Technique**	**Pathogen application**	**Ref.**
Real-time PCR	*Mycobacterium avium* subsp. *Paratuberculosis*	[15]
	*E. coli* O157:H7	[16]
	*S. aureus*	[17]
	*L. monocytogenes*	[8,18]
	*S. enterica* serovar *typhimurium*	[19]
Multiplex PCR	*E. coli* O157:H7; *Salmonella* spp.; *Shigella* spp.	[20]
	*L. monocytogenes* and *Salmonella* spp.	[21]
	*Campylobacter* spp.*, Salmonella* spp., *E. coli, Shigella* spp., *Vibrio cholerae, Y. enterocolitica*	[22]
Reverse transcriptase PCR	*E. coli* O157:H7	[23]
	*E. coli* O157:H7, *V. cholerae, S. typhi*	[24]
Immuno PCR	*Streptococcus pyogenes*	[25]
	*E. coli* shiga-toxin 2	[26]
NASBA	*L. monocytogenes*	[12,13]
	*Campylobacter* spp., *L. monocytogenes, S. enterica* serovar *Enteritidis*	[27]

**Table 4.** Immunosensor-based detection of selected bacterial pathogens. Key: [C] - capture antibody; [S] - secondary antibody; [D] - detection antibody.

**Bacterial strain**	**Biosensor format**	**Assay format**	**Antibodies**	**Sensitivity**	**Ref.**
*B. anthracis*	Optical	Sandwich	Biotinylated rabbit anti-*B. anthracis* polyclonal [C]; rabbit anti-*B. anthracis* polyclonal CY5 [D]	3.2 × 10^5^ spores/mg powder	[108]
	Piezoelectric	Capture	Rabbit polyclonal anti-*B. anthracis* [C]	333 spores/mL	[109]
*B. globigii*	Optical	Sandwich	Goat anti-*B. globigii* [C]; rabbit anti-*B. globigii* [S]; goat anti-rabbit-AP [D]	1 spore	[110]
*B. subtilis*	Potentiometric	Sandwich	Biotinylated polyclonal anti-*B. subtilis* antibody [C]; fluorescein-labelled polyclonal anti-*B. subtilis* antibody [S]; anti-fluorescein urease-conjugated antibody [D]	3 × 10^3^ spores/mL	[111]
*F. tularensis*	Magnetic	Sandwich	Monoclonal anti-*F. tularensis* [C]; biotinylated monoclonal anti-*F. tularensis* on streptavidin-coated magnetic beads [D]	1 × 10^4^ – 1 × 10^6^ CFU/mL	[112]
*M. tuberculosis*	Piezoelectric	Capture	Rabbit anti-*M. tuberculosis* [C]	1 × 10^5^ cells/mL	[113]
	Voltammetric	Sandwich	Biotinylated rabbit anti-*M. tuberculosis* [C]; murine monoclonal anti-*M*. tuberculosis [S]; rabbit anti-mouse-AP [D]	1.0 ng/mL	[114]
*N. meningitidis*	Optical	Direct	Murine anti-group C polysaccharide [C]	-	[115]
*V. cholerae*	Amperometric	Sandwich	Rabbit polyclonal anti-*V. cholerae* [C]; mouse anti-*V. cholerae* [S]; anti-mouse AP [D]	1 × 10^5^ cells/mL	[116]
	Optical	Capture	Monoclonal anti-*V. cholerae* O1 [C]	1 × 10^5^ – 1 × 10^9^ cells/mL	[117]
*Y. pestis*	Magnetic	Sandwich	Monoclonal anti-F1 antigen [C]; biotinylated monoclonal anti-F1 on streptavidin-coated magnetic beads [D]	2.5 ng/mL antigen	[118]

**Table 5.** A selection of immunosensor-based assays for viral pathogen detection. Key: [C] - capture antibody; [P] - primary antibody; [S] - secondary antibody; [D] - detection antibody. Where primary antibodies are used, the antigen/epitope is immobilised on the sensor surface.

**Virus**	**Biosensor platform**	**Assay format**	**Antibodies**	**Ref.**
Herpes simplex virus (HSV) 1 and 2, Varicella-Zoster virus (VSV), Cytomegalovirus (CMV) and Epstein-Barr virus (EBV)	Piezoelectric	Capture	Mouse monoclonal antibodies to herpes simplex virus 1 and 2, cytomegalovirus, Epstein-Barr virus and Varicella Zoster virus [C]	[131]
Foot and mouth virus (FMV)	Impedimetric	Indirect	Murine monoclonal [P]	[132]
African swine fever virus (ASF)	Piezoelectric	Capture	Murine monoclonal [C]	[133]
Bovine diarrhoeal virus (BVD)	Optoelectronic	Capture	Anti-BVD monoclonal	[134]
Cymbidium mosaic potexvirus (CymMV) and Odontoglossum ringspot tobamovirus (ORSV)	Piezoelectric	Capture	Rabbit polyclonal	[135]
SARS-associated coronavirus (SARS-CoV)	Piezoelectric	Capture	Horse polyclonal anti-SARS-CoV [C]	[136]
Human immunodeficiency virus (HIV-1)	Piezoelectric	Capture	Murine anti-trans activator of transcription (TAT) HIV [C]	[137]
Hepatitis C virus (HCV)	Optical	Indirect	Polyclonal IgG antibodies [P]; Polyclonal goat anti-human IgG-HRP [D]	[138]
Cowpea mosaic virus (CPMV)	Optical	Capture	Anti-CPMV recombinant antibody (scFv) fused to the constant light chain (C_L)_ domain containing a C-terminal cysteine residue [C]	[139]
Ebola virus (EBOV)	Optical	Capture	Mouse monoclonal anti-EBOV [C]	[140]
	QCM	Capture	Rabbit polyclonal antibody [C] or Mouse monoclonal antibody [C]	
Avian leucosis virus (ALV)	Optical	Capture	Monoclonal anti-ALV-J	[141]
Rift valley fever virus (RVF)	Fibre optic immunosensor	Sandwich	Mouse polyclonal anti-RVF [C]; Polyclonal IgG antibodies [S]; Goat anti-human IgG – HRP [D]	[142]
West Nile virus (WNV)	Amperometric	Indirect	Polyclonal IgG antibodies [P]; Goat anti-human IgG-HRP [D]	[143]

**Table 6.** A selection of immunosensor-based assays for marine algal toxin detection. Key: [C] - capture antibody; [P] - primary antibody; [D] - detection antibody; LOD - limit of detection.

**Toxin**	**Biosensor Format**	**Assay Format**	**Antibodies**	**LOD**	**Ref**
Brevetoxin	Amperometric	Indirect	Goat-anti brevetoxin [P]	15 μg/L	[150]
Domoic acid (DA)	Amperometric	Indirect	Sheep polyclonal [P]; anti-sheep IgG-AP [D]	2 μg/L	[151]
	Amperometric	Indirect	Rabbit polyclonal [P]	0.1 μg/L	[152]
	Optical	Indirect	Monoclonal anti-DA [P]	1.8 μg/L	[153]
	Optical	Indirect	Monoclonal anti-DA [P]	0.1 μg/L	[154]
	Optical	Indirect	Rabbit polyclonal anti-DA [P]	3 μg/L	[155]
Microcystin-LR (MC)	Optical	Direct	Monoclonal anti-MC-LR-Cy5 [P]	0.03 μg/L	[156]
	Capacitance	Capture	Monoclonal anti-MC-LR [C]	7 pg/L	[157]
	Optical	Direct	Monoclonal anti-MC-LR-Cy5 [P]	30 ng/L	[158]
Okadaic acid (OA)	Optical	Direct	Mouse monoclonal anti-OA-HRP [P]	0.1 μg/L	[159]
	Amperometric	Direct	Mouse monoclonal anti-OA-AP [P]	1.5 μg/L	[151]
	Piezoelectric	Capture	Monoclonal anti-OA [C]	3.6 μg/L	[160]
	Amperometric	Capture	Monoclonal anti-OA [C]	2 μg/L	[161]
	Amperometric	Indirect	Mouse monoclonal anti-OA [P]; goat anti-mouse-HRP or AP [D]	0.03 μg/L	[162]
Saxitoxin (STX)	Amperometric	Direct	Donkey anti-STX-glucose oxidase [P]	2 μg/L	[150]
